# Evaluation of a fertility awareness-based shared decision-making tool part 1: Study design and impact on clinician knowledge

**DOI:** 10.1016/j.pecinn.2022.100061

**Published:** 2022-06-28

**Authors:** Marguerite Duane, Virginia Martinez, Meghan Berry, Michael D. Manhart

**Affiliations:** a FACTS, 1020 Kearny St NE, Washington DC 20017, USA; b Couple to Couple League International, 5440 Moeller Avenue Suite 149, Cincinnati, OH 45212, USA

**Keywords:** Shared decision making, Fertility awareness, Family planning, Medical knowledge

## Abstract

**Objective:**

To assess the impact of a Shared Decision-Making (SDM) tool for fertility awareness-based methods (FABMs) of family planning.

**Methods:**

Clinicians familiar with at least one FABM were randomly invited to participate in a prospective cross-over study to compare usual practice to the use of the SDM tool when discussing FABMs with patients. Patients completed surveys pre- and post-office visit and six months later. The primary outcome explored the effect of online education on use of the SDM tool on clinicians' knowledge of FABMs.

**Results:**

Of 278 clinicians contacted, 54% could not be reached, and 15% did not provide women's health services. The 26 clinicians enrolled were experienced, with more than half recommending FABMs for ≥10 years, and 73% recommending more than one FABM to patients. Knowledge scores significantly improved after online training and use of the SDM tool (baseline mean score = 9.54 (scale of 0–12); post-training mean score = 10.73, *p* < 0.002).

**Conclusions:**

Education about FABMs and training on use of the SDM tool improved knowledge scores even among an experienced cohort of clinicians.

**Innovation:**

The novel SDM tool can better equip clinicians to meet the rising patient interest in FABMs.

## Highlights

• Shared decision-Making (SDM) tools and additional training for clinicians help patients make informed choices.
• A decision-making tool focused on fertility awareness based method (FABM)selection was developed and tested
• Online education and training on use of the SDM tool significantly improved clinicians’ knowledge of FABMs.
• This novel tool can better equip clinicians to meet rising patient interest in FABMs.

## 1 Introduction

Shared decision-making tools improve a patient's knowledge regarding available treatment options and can facilitate a more active role in their health care [1]. Research shows women prefer to engage in a shared decision-making process when choosing a family planning method to prevent pregnancy, and such a client-centered approach has also been shown to increase satisfaction with and continued use of the method [[2], [3], [4]]. This approach may be preferable for family planning because it bridges the gap between attributes that matter most to patients (fewer side effects) and what clinicians perceive is most important (effectiveness rates) [3].

Fertility awareness-based methods of family planning (FABMs) use the physiological signs or biomarkers of a woman's cycle—cervical fluid secretions, basal body temperature, and/or urinary hormones—to assist her in identifying the fertile window: the days in her cycle when she can become pregnant [5]. Using this information, couples can choose whether or not to engage in sexual relations based on their family planning goals. Individual FABMs require tracking different biomarkers, have different levels of effectiveness [6,7] and distinct learning curves [8]. Unfortunately, most physicians have limited knowledge about the different evidence-based FABMs and their effectiveness rates, advantages and disadvantages [[9], [10], [11], [12], [13]]. As a result, most clinicians may not be able to provide accurate information to patients who may be interested in these methods.

When compared to usual care, the evidence shows that decision aids can improve people's knowledge regarding options and can encourage them to take a more active role in the decision-making process [14,15]. When choosing a method of family planning, inadequate decision support may lead women or couples to choose a method that is not well suited for them, which may lead to dissatisfaction, discontinuation, or incorrect use of a method. Aware of this and the paucity of decision aids for family planning that include FABMs, our group sought to develop and test the impact of a Shared Decision-Making tool for FABMs.

Herein, we describe the development and pilot testing of the SDM tool, and the protocol used to evaluate its impact during the clinician-patient interaction compared to normal practice; we also report on the enrollment of clinicians in the study and the impact of the interventions on their knowledge of FABMs. A subsequent publication will focus on the utility of the SDM tool for patients considering FABMs for family planning.

## 2 Methods

### 2.1 Development and piloting of the shared decision-making tool

We first developed and pilot tested the Shared Decision-Making Tool. Five evidence-based FABMs were selected for inclusion in the Shared Decision-Making tool based on the evidence for their effectiveness to prevent pregnancy and expectation of familiarity across the United States. The five methods chosen were the Billings Ovulation Method, the Creighton Model, the Marquette Model, the Standard Days Method, and the Sympto-Thermal Method. Information about each method included a brief description of how it works, effectiveness rates to avoid pregnancy, advantages, challenges, cost, time to learn, and links for further information.

**Fig. 1:**
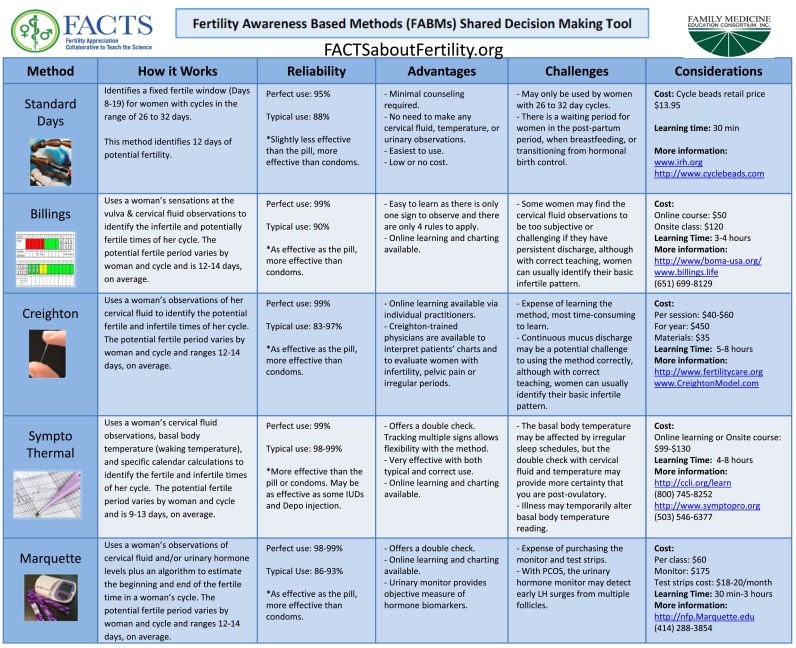
FACTS shared decision-making tool. Five evidence-based FABMs are compared by discussing how they work, their reliability to avoid pregnancy, advantages, and challenges. The tool facilitates a patient-centered dialogue between clinician and patient to promote informed decision making in selecting a patient-appropriate FABM.

Three focus groups were conducted among reproductive-aged women and couples to pilot and optimize the tool and the information provided. The resulting prototype was reviewed by 10 experts on the various methods, two for each method, to ensure the information provided was accurate. The tool was then piloted with three physicians who used it to guide the discussion about options for family planning with a total of 20 patients interested in using FABMs. The patients were asked a series of questions about the tool, its ease of use, helpfulness in selecting a method, and recommendations for improvement. The pilot study was reviewed and approved by the Georgetown School of Medicine IRB. The final instrument is a single page graphic measuring 8.5 × 11 in (Fig. 1).

### 2.2 Recruitment of clinicians

To test the impact of the SDM Tool in comparison to usual practice, clinicians were selected at random from an aggregated list of clinicians from across the United States who were already familiar with or knowledgeable about at least one natural method. FABM-trained or knowledgeable clinicians were identified via their affiliation with FABM organizations and/or public natural family planning (NFP)/FABM listservs or databases to generate one master list of names with all duplicates removed. For this study, a clinician was defined as a physician (MD, DO), physician assistant, midwife, or nurse practitioner.

A total of 570 clinicians were identified from these lists and stratified into one of seven groups based on the FABM the clinician knew and used (i.e., one of five FABMs, multiple methods, or unknown). Potential participants were then randomly selected within each stratum and invited to participate via email and/or phone follow up.

**Fig. 2:**
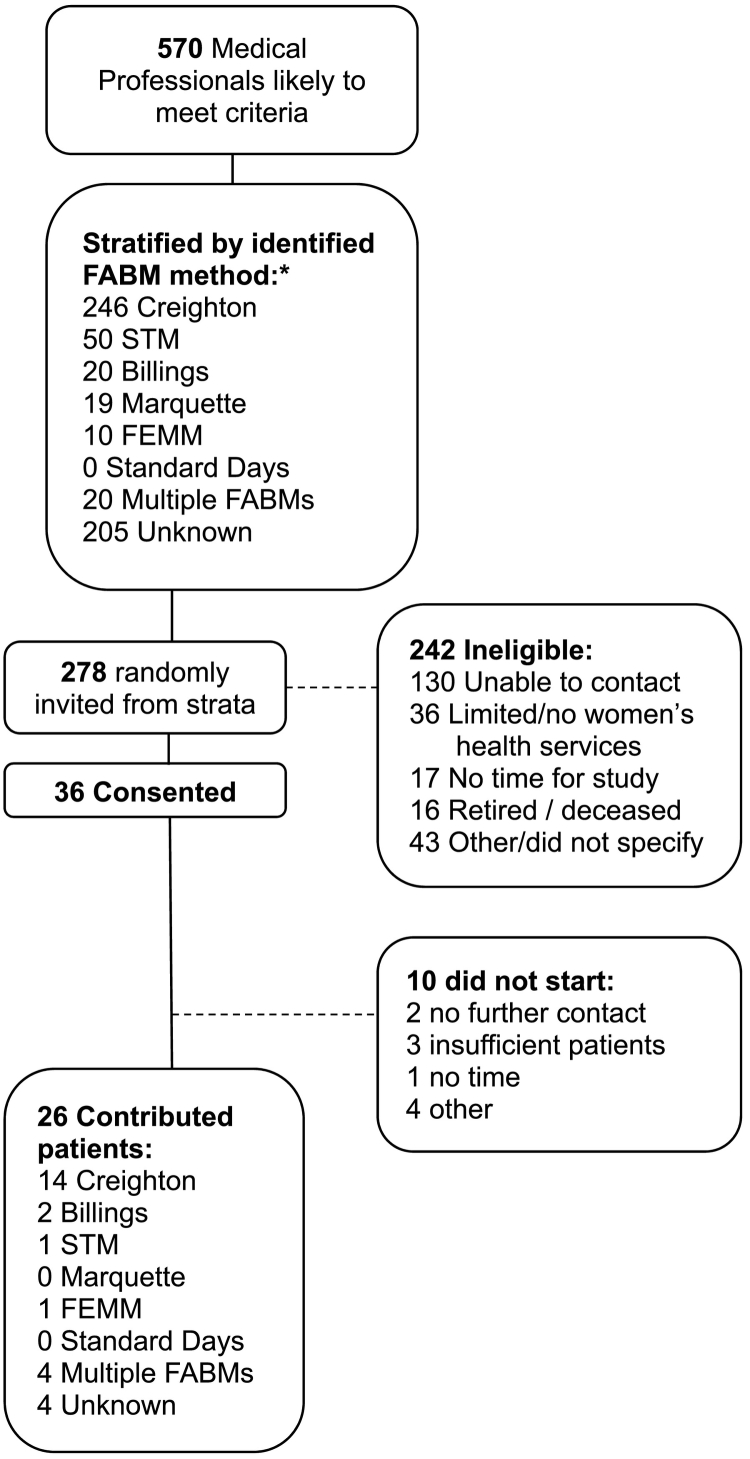
Clinician recruitment. A collated list of 570 clinicians was stratified by type of FABM of which each professional had knowledge. Out of 278 names randomly selected from the strata and invited to participate, 36 consented, with 10 not participating beyond initial consent. The reasons for declining are listed, and the strata from which the 26 clinician participants were drawn are illustrated.

Clinicians were invited to participate if they (i) were already recommending at least one FABM as a family planning option to some of their patients, (ii) anticipated discussing FABMs with at least 10 patients during a 6-month period, and (iii) were willing to learn about all evidence-based FABMs and make use of the SDM tool provided. Recruitment continued until at least 30 clinicians consented to participate. Fig. 2 illustrates the selection and enrollment of the participating clinicians.

### 2.3 Control period

**Fig. 3:**
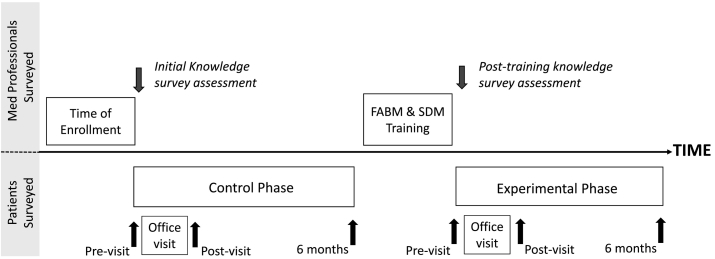
Study design. Queries and interventions of clinician participants are illustrated above the line while those of patient participants are illustrated below the line.

The study was approved by and conducted under the Georgetown School of Medicine IRB. We employed a simple crossover design to evaluate the impact of the SDM tool (Fig. 3). After providing consent, the clinicians completed a knowledge assessment to gauge current familiarity with FABMs. Eligible control patients were invited to participate in the study for a period of up to six months or until 10 patients were entered, whichever came first. During this control period, clinicians employed their usual counseling practices when discussing FABMs during office visits with enrolled patients.

### 2.4 Recruitment of patients

Female patients were invited to participate if they were 18 to 44 years of age and were presenting for an office visit scheduled as: well woman, new patient, postpartum, family planning, NFP counseling, or OB-family planning visit. Patients also needed to indicate they had started a new family planning method in the previous 6 months or were considering starting, changing, or re-starting an FABM in the next 6 months. Invited women were enrolled if they consented to the study, agreed to complete pre- and post-office visit surveys at the time of the appointment and to be contacted 6 months later to complete an online follow-up survey.

### 2.5 Experimental period

Upon completion of the control period, participating clinicians watched a short (7 min) training video to demonstrate implementation of the SDM tool during an office visit. After watching the online training video, each clinician attended a one-hour CME presentation that provided an overview of all the evidence-based FABMs and completed the same knowledge assessment administered at the start of the control period.

Once the clinician completed these steps, their experimental period began. During the office visit of a consented patient, the decision-making tool was employed to help guide the conversation with the patient. As in the control period, consented patients completed a short survey prior to and immediately after the office visit as well as a six-month follow-up survey. The experimental period continued for at least as long as the control period for that clinician or until 15 patients were enrolled.

### 2.6 Analysis

The primary outcome of the study explored the effect of online education and training on use of the SDM tool on clinicians' knowledge of FABMs. This was measured first by comparison of pre- and post-training scores on the FABM knowledge quiz and then by an increase in the number of different methods recommended to patients in the experimental period compared to control. We estimated a minimum 12.5% mean increase in pre- to post-test knowledge scores. With 20 physicians completing the study and a one-sided Type I error rate of 0.05, the study had 99% power to reject the null hypothesis of no improvement in pre- to post-test score.

To evaluate the potential increase in the number of different FABMs recommended when using the SDM tool, we assumed most clinicians recommend only one FABM and some recommend two, resulting in a baseline mean of 1.36 (standard deviation 0.7). With 20 clinicians completing the study, we had a 92% chance of detecting an average increase of 0.5 in the number of FABMs recommended, a 99.9% likelihood of detecting an increase of one.

The secondary outcome explored the usefulness of the SDM tool for patients. We hypothesized a superior “fit” of the particular FABM recommended to a patient when the SDM tool was employed. This was measured using patient satisfaction scores from the post-office visit survey, and for patients starting or changing FABMs, the level of “fit” was measured by assessing continued use at the 6-month follow-up survey.

For this study, we assumed that prior to training on use of the SDM tool, 25% of women who discussed FABMs with their clinician would adopt an FABM and continue to use that method for at least six months. We hypothesized that for patients who experienced use of the SDM tool by a trained clinician, as many as 35% of women would adopt an FABM and continue its use for at least six months. With a two-sided Type I error rate and 80% power to reject the null hypothesis of no difference in the proportion of women still using adopted FABMs after 6 months, we would need 155 women in each of the control and experimental groups to detect a significant difference in proportions.

## 3 Results

### 3.1 Pilot testing of SDM Tool

Pilot testing revealed that 94% of patients found the tool “very easy” or “easy to use.” Seventy-four percent stated the tool was “very helpful” or “helpful” to them, and the remaining felt it was “somewhat helpful.” Qualitative analysis of the focus group comments revealed that patients most liked the side-by-side comparisons, the clear and concise descriptions, readability, and ease of use. Women least liked that the tool seemed compressed and lacked color visuals. From the pilot testing, we concluded the tool had met its design objectives and was ready for testing in the full protocol.

### 3.2 Clinician recruitment

Over a period of approximately one year, 278 clinicians nationwide were contacted to invite them to participate in the study. Despite recruiting from lists of clinicians already familiar with and recommending FABMs, 242 of those contacted (87%) were ineligible or declined to participate. Most clinicians (54%, *n* = 130) were ineligible simply because they could not be reached via publicly available contact information. Fifteen percent (*n* = 36) declined because they provided limited or no women's health services, 18% (*n* = 43) declined for other reasons, and 13% declined for lack of time or due to retirement/death (Fig. 2).

In total, 36 clinicians consented to participate in the study. For various reasons, 10 of them contributed no data; four had institutional conflicts that prevented participation, three could not identify patients who met enrollment criteria, two were lost to follow up after consenting, and one had insufficient time to enroll patients. A total of 26 clinicians contributed patients in both the control and experimental phases of the study.

Of the 26 clinicians participating in the study, 24 were physicians (19 MD, 5 DO); nine specialized in obstetrics and gynecology (OB/GYN), nine in family medicine, and six stated they were in family medicine with an OB emphasis. A midwife and a nurse practitioner were also part of the clinician cohort. The professional cohort recruited was experienced in FABMs, with 92% reporting they already teach, recommend, or refer patients for FABMs, and >50% reporting they have recommended FABMs for 10 years or longer.

The recruited cohort was also more diverse in their training and familiarity with different FABMs than suggested by the methods used to stratify for enrollment. Prior to the study, most clinicians (73%) taught, recommended, or referred patients to learn more than one FABM.) On average, this cohort recommended 2.5 different FABMs at baseline, which is significantly more than the 1.4 anticipated when sizing the study to measure an increase in number of FABMs recommended after learning how to use the SDM tool. When asked to indicate the specific methods to which they had referred patients, the results were as follows: the Creighton Model (22), the Sympto-Thermal Method (16), the Billings Ovulation Method (13), the Marquette Model (12), the Fertility Education and Medical Management (FEMM) (3), and the Standard Days Method (3). Finally, it is noteworthy that six of the 26 clinicians (23%) took maternity leave between their control and experimental time periods, which delayed patient recruitment.

### 3.3 Clinician knowledge

**Fig. 4:**
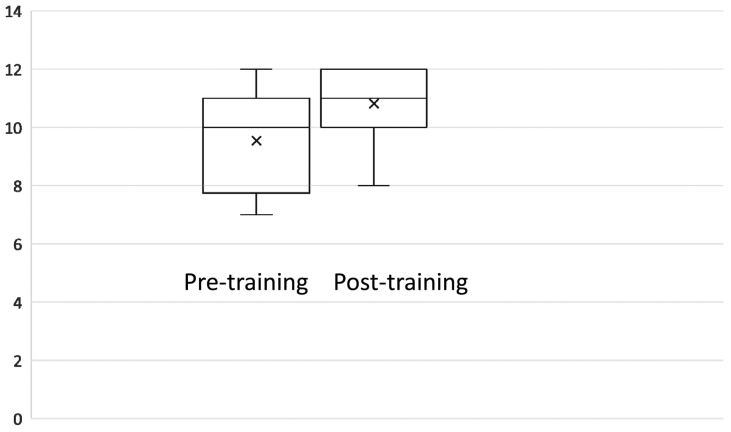
Clinician knowledge scores. Box & Whisker plot of mean knowledge scores (test score range 0–12) before and after training on the SDM tool. An ‘x’ within a box indicates mean group values. Boxes represent upper and lower quartiles; whiskers indicate scores outside the quartile limits. The mean score post training was significantly higher (*p* < 0.002) than the pre-training scores.

At baseline, the mean knowledge test score was 9.54 (on a scale of 0–12). Two physicians received a perfect score, and another 10 physicians answered 10 or 11 questions correctly, reflecting the recruited cohort's experience with FABMs. After education and training on the SDM tool, 80% of clinicians increased their test scores, resulting in a mean of 10.73 (*p* < 0.002 vs. baseline); 10 earned a perfect score, and another 15 answered 10 or 11 questions correctly (Fig. 4).

## 4 Discussion and conclusion

### 4.1 Discussion

The primary outcome of this study explored the effect of online education and training on use of the SDM tool on clinicians' knowledge of FABMs. The results demonstrate that a brief educational intervention that includes a 1-h CME presentation can significantly increase physicians' knowledge of FABMs. Since this sample of clinicians already demonstrated high baseline knowledge, we might expect to see even larger knowledge gains among clinicians with limited FABM familiarity.

A surprising finding was the unexpectedly high number of different methods recommended by the recruited cohort as part of their usual practice prior to the study. For this reason, we could not document whether their improved knowledge translated directly to a significant increase in the number of different FABMs discussed or recommended to patients once the SDM tool was implemented. Initially, we predicted the clinicians in the study would recommend, on average, 1.4 FABMs with a standard deviation of 0.7. Yet, at baseline, the clinicians in the study recommended an average of 2.5 methods with a standard deviation of 1.4. This substantially reduced the study's power to detect an average increase of 0.5 FABMs recommended from 0.93 to 0.48. The secondary outcome of the study—usefulness of the SDM tool for patients considering FABMs—may shed light on this and will be the subject of a subsequent publication.

Notably, clinicians trained in FABMs were difficult to find despite having access to multiple lists of clinicians and educators for several different FABMs. The challenge of simply identifying clinicians knowledgeable about FABMs limits women's access to these family planning options they may desire. Of particular concern was the high proportion of clinicians listed as “knowledgeable in FABMs” who did not provide care for women of reproductive age or could not be contacted due to outdated or incorrect information. Beyond this, even if all 570 clinicians initially identified for the study offered FABMs, this would represent less than 0.5% of all practicing OB/GYN physicians (18,590) and family physicians (98,590) in 2018 [16].

The fact that less than 0.5% of OB/GYN and family physicians are identifiable as knowledgeable about FABMs presents a significant barrier to access for women and couples interested in using these methods. Over the last several decades, the drive to increase access to reproductive health services has led to increased public funding and administration of what is considered highly or moderately effective modern contraceptive care, including sterilization and hormonal methods [17]. According to data from the National Survey of Family Growth over the last 15 years, approximately 90% of women at risk for unintended pregnancy currently use a method of contraception [18]. Notably, the percentage of women not using any contraception remained steady during the same time period despite hormonal contraceptive methods being readily offered at private practices, family planning clinics, and health departments [18]. There has also been a moderate increase in women using FABMs, suggesting that women are looking for alternatives to conventional contraceptive methods, and that FABMs could potentially fill the unmet need for family planning [18]. Despite the evidence for women's growing interest in FABMs along with data documenting similar effectiveness rates for some FABMs and various hormonal contraceptive methods, the effort to increase family planning services has not included access to or increased cost coverage for FABMs, potentially limiting their use.

The lack of access to FABM-trained clinicians likely reflects the fact that most clinicians receive minimal education about these methods and remain uninformed about modern, evidence-based FABMs [10]. This lack of exposure and education persists despite significant progress in knowledge about FABMs and their clinical applications in reproductive health over the last four decades. According to Stanford et al., more than half of the primary care physicians they surveyed indicated they do not mention FABMs, or mention them with reservations, when patients inquire about family planning options [11]. Furthermore, the survey found most physicians are misinformed about the effectiveness of modern FABMs, with many only discussing the calendar rhythm method, which was developed in the 1930s and is now considered outdated. More recent studies of Canadian physicians and residents [9], as well as providers of family planning services from Title X clinics [19], indicate knowledge and perceptions about FABMs have not progressed. Similarly, an unpublished survey of 650 medical students from seven different U.S. medical schools revealed that 80–90% of students are not familiar with any of the modern, evidence-based FABMs. On the other hand, 60–70% of students are interested in learning about these methods, with two-thirds of them expressing interest in online learning opportunities [20].

Even though the Affordable Care Act (ACA) expanded access to other contraceptive methods, access to FABMs remains limited despite rising interest [21,22]. Studies indicate that over the last two decades, women's interest in using FABMs for family planning has increased [[23], [24], [25]]. Simultaneously, there has been an explosion of mobile health applications (apps) available for women to track their fertility, with over 500 apps on the market. Other than activity tracking apps, fertility apps are the most frequently downloaded health app in the Apple app store [26]. A recent survey employing a nationally representative sample of U.S. adults revealed that fertility trackers are used at least once a month by 19% (± 4%) of respondents [27]. This suggests regular fertility tracker use is comparable to the 16% of women currently using oral contraception [28]. Unfortunately, most of these apps do not integrate evidence-based FABM guidelines and, thus, may not be sufficient to help couples successfully prevent pregnancy [29]. Hence, there is a need for comprehensive clinical resources to support clinicians in providing their patients access to accurate, evidence-based information for various types of modern FABMs, to meet the rising interest in these methods.

A strength of this study is its comparative design and implementation in non-academic medical settings. Each clinician's utilization of the SDM tool can be evaluated against his or her usual practice, and the cohort recruited reflects medical settings commonly experienced by patients. One might also argue that the brief training administered with the tool, having demonstrated significant improvement in knowledge among a cohort experienced in FABMs, would have an even larger effect on clinicians with less knowledge about these methods.

A study weakness is its lack of power to show an increase in the number of FABMs recommended by clinicians following integration of the SDM tool. In declining to participate, some clinicians explained they had committed to recommend a single FABM and were thus not interested in using a Shared Decision-Making tool to select among different FABMs. Others who declined to participate noted they were not willing to use a Shared Decision-Making tool in patient care that included options they would not recommend or did not offer in their practice. Exclusion of several single-FABM recommenders would introduce an upward bias in the study data on the number of FABMs recommended by clinicians.

### 4.2 Innovation

This study applies the well-established concept of shared decision-making to a novel area of family planning where there is a lack of knowledge among clinicians and lack of resources for clinical support. Shared decision-making tools have been developed for women to choose among conventional methods of birth control [4]. However, such resources rarely include modern fertility awareness-based methods of family planning, even though some FABMs have effectiveness rates for pregnancy prevention comparable to other behavioral methods of birth control [6,7]. This is the first effort, performed in a non-academic setting, at evaluating a decision tool created specifically for FABMs. Since this tool contains detailed information about FABMs, their effectiveness rates, advantages and challenges, clinicians less familiar with FABMs may use the tool at every well women exam, family planning or post-partum visit to offer more family planning options to patients. An updated version of the SDM tool based on the most up to date evidence as well as a 1 h CME webinar on FABMs may be accessed via the FACTS website.

Limited access to FABM-trained clinicians when women's interest in reproductive health monitoring is growing—as evidenced by the high rate of fertility tracker use—points to the need for simple decision-making aids and additional training for physicians and other clinicians to help patients make informed choices. Since effectiveness studies of FABMs to prevent pregnancy have been done almost exclusively with people who learn these methods from trained instructors, a physician's familiarity with the different evidence-based methods and the availability of local certified teachers will facilitate more appropriate referrals.

### 4.3 Conclusion

Notwithstanding these limitations, integration of the SDM tool was well received by clinicians and added to their already high knowledge level of FABMs. Implementation of the online educational presentation would be an effective way to increase the number of clinicians knowledgeable about these methods. Such education about modern evidence-based fertility awareness-based methods would enable clinicians to offer them to interested patients. Future work will focus on examining patient reactions to the SDM tool introduced in this study.

## Declaration of Competing Interest

The authors declare that they have no known competing financial interests or personal relationships that could have appeared to influence the work reported in this paper.
